# Glutamine transporters as effective targets in digestive system malignant tumor treatment

**DOI:** 10.32604/or.2024.048287

**Published:** 2024-09-18

**Authors:** FEI CHU, KAI TONG, XIANG GU, MEI BAO, YANFEN CHEN, BIN WANG, YANHUA SHAO, LING WEI

**Affiliations:** 1 School of Chinese Materia Medica, Guangdong Pharmaceutical University, Guangzhou, 510006, China; 2 Department of Pharmacy, Tianjin Medical University General Hospital, Tianjin, 300070, China

**Keywords:** Glutamine transporter, Targeted therapy, Inhibitors, Cancer

## Abstract

Glutamine is one of the most abundant non-essential amino acids in human plasma and plays a crucial role in many biological processes of the human body. Tumor cells take up a large amount of glutamine to meet their rapid proliferation requirements, which is supported by the upregulation of glutamine transporters. Targeted inhibition of glutamine transporters effectively inhibits cell growth and proliferation in tumors. Among all cancers, digestive system malignant tumors (DSMTs) have the highest incidence and mortality rates, and the current therapeutic strategies for DSMTs are mainly surgical resection and chemotherapy. Due to the relatively low survival rate and severe side effects associated with DSMTs treatment, new treatment strategies are urgently required. This article summarizes the glutamine transporters involved in DSMTs and describes their role in DSMTs. Additionally, glutamine transporter-target drugs are discussed, providing theoretical guidance for the further development of drugs DSMTs treatment.

## Introduction

Malignant tumors pose a substantial threat to human health and their incidence is steadily increasing. Recently, research on the metabolic mechanisms of tumors, with a particular focus on glutamine (Gln) metabolism, has emerged as a prominent area of study. Gln, a non-essential amino acid that is abundant in the human body, is primarily acquired intracellularly via Gln transporter self-synthesis and uptake. In cancer cells, a substantial demand for Gln arises due to their rapid proliferation and growth, resulting in Gln-dependence. This dependence highlights the crucial role of Gln transporters in Gln metabolism. Consequently, targeting these transporters is a promising approach for tumor treatment. Digestive system malignant tumors (DSMTs) manifest in organs, such as the intestines, esophagus, stomach, pancreas, and liver. Notably, esophageal, gastric, hepatocellular, and colorectal cancers rank among the top ten tumors causing substantial morbidity, with all five types of DSMTs featuring the top 10 mortality rates [1]. Therefore, DSMTs pose a substantial threat to human health worldwide. The current clinical treatments for DSMTs primarily involve surgery, radiotherapy, and cancer immunotherapy. However, these treatments often induce adverse reactions and encounter challenges related to multidrug resistance. This article aims to provide a comprehensive overview of the role of Gln transporters in DSMTs, along with an exploration of targeted therapeutic drugs, with the goal of providing valuable insights into the drug discovery process for DSMTs therapy and deepening our understanding of the underlying mechanisms.

## Gln and Gln Transporters

### Function of Gln

Gln is recognized as the most abundant non-essential amino acid in human plasma [2], with a concentration reaching 0.8 mM under physiological conditions and the potential to comprise up to 40% of the total intracellular amino acids content [3]. In healthy cells, Gln serves as a coding amino acid for protein synthesis, which is crucial for sustaining cell growth, and also acts as a versatile amino acid with essential roles in cellular metabolism and homeostasis. First, Gln undergoes catalysis by glutaminase (GLS), generating glutamate (Glu), which contributes to the tricarboxylic acid (TCA) cycle by producing alpha-ketoglutarate (α-KG), a carbon source for synthesizing lipids, glucose, and nucleotides [4]. Second, the amide and amino groups of Gln act as nitrogen sources for the synthesis of non-essential amino acids, nucleotides, and amino sugars, which are vital for cell proliferation [5,6]. Third, Gln plays a critical role in glutathione (GSH) synthesis when combined with cysteine (Cys) and glycine to maintain cellular oxidative-reductive balance [7]. Last, Gln enters cells by exchange with leucine (Leu) and activates the mammalian target of rapamycin (mTOR) [8], which further regulates lipid synthesis, nucleotide synthesis, and glucose metabolism. In cancer cells, the demand for Gln increases and its metabolism is often rewired to meet the unique metabolic requirements associated with tumorigenesis and rapid proliferation. Cancer cells heavily rely on Gln as a major carbon and nitrogen source for survival, because aerobic glycolysis is the main energy source. Gln is catabolized via glutaminolysis to yield metabolites crucial for cell growth and proliferation. For instance, Gln replenishes the TCA cycle, which is hindered by the Warburg effect [9], by converting into α-KG. This α-KG can then enter the TCA cycle to facilitate energy production and other anabolic reactions [10]. Glutaminolysis, a vital metabolic process in cancer cells that involves the breakdown of Gln, is essential for the synthesis of biomacromolecules necessary for supporting biosynthetic requirements, including proteins, fatty acids, and nucleotides. Moreover, cancer cells often cannot synthesize sufficient Gln internally, leading to a “glutamine-dependent phenomenon” in which high Gln levels sustain normal cancer cell growth, whereas low Gln environments induce cell growth arrest. Additionally, oncogenes, notably the Kirsten rat sarcoma virus (*KRAS*), reprogram Gln metabolism to support cancer cell growth. Downstream molecules, such as c-Myc, activate genes related to Gln catabolism, such as *GLS*, thereby promoting Gln uptake and signaling pathways crucial for cancer cell proliferation [11]. Overall, while Gln plays an essential role in normal cellular functions, its metabolism is altered in cancer cells to meet the heightened metabolic demands linked to tumorigenesis and proliferation. Understanding these alterations in Gln metabolism is crucial for the development of targeted therapies to disrupt cancer cell metabolism and inhibition of tumor growth.

### Gln transporters

**Figure 1:**
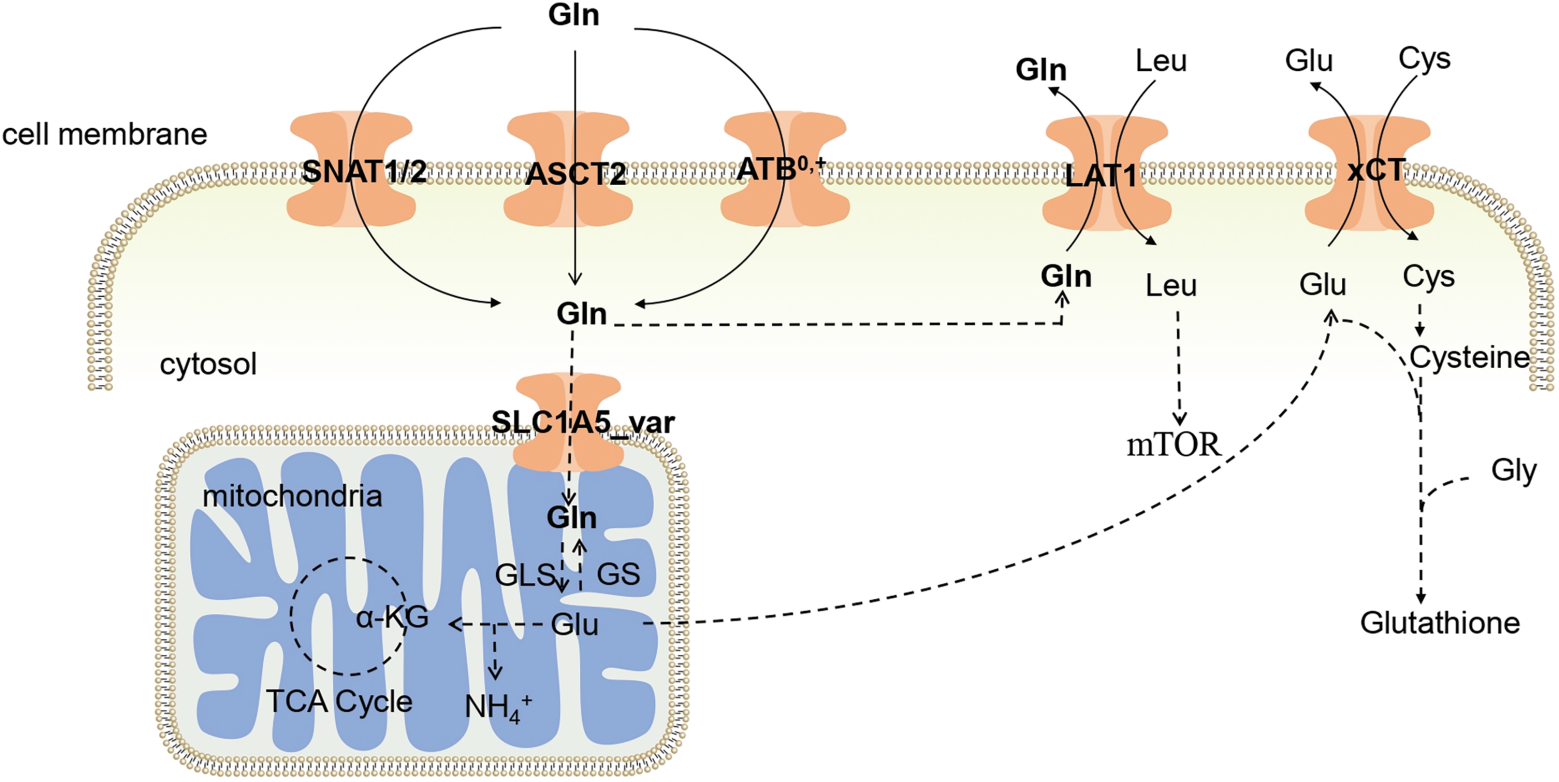
Glutamine uptake in tumors. Gln enters cells through the phospholipid bilayer of cells by ASCT2, ATB^0,+^, and SNAT1/2, and part of Gln enters mitochondria, where it is catalyzed by GLS to generate Glu, thereby participating in the TCA cycle. The Glu produced in the mitochondria is interchangeable with Cys through xCT, helping Cys enter the body to synthesize Glutathione. ASCT2: Alanine-serine-cysteine transporter 2; ATB^0,+^: Amino acid transporter B^0,+^ ; LAT1: *L*-type amino acid transporter 1; xCT: Cystine transporter; SNAT1: Sodium-coupled neutral amino acid transporter 1; SNAT2: Sodium-coupled neutral amino acid transporter 2; Gln: Glutamine; Glu: Glutamate; Leu: Leucine; Cys: Cysteine; mTOR: Mammalian target of rapamycin; Gly: Glycine; GLS: Glutaminase; GS: Gln synthetase; α-KG: α-ketoglutarate; TCA Cycle: Tricarboxylic acid cycle.

As a hydrous amino acid, Gln cannot permeate the cell membrane directly, and requires specific membrane transporters to facilitate its passage through this barrier. Consequently, Gln transporters play a pivotal role in determining the ability of cells to utilize Gln. Gln transporters in the body encompass the alanine-serine-cysteine transporter 2 (ASCT2), amino acid transporter B^0,+^ (ATB^0,+^), *L*-type amino acid transporter 1 (LAT1), cystine/glutamate antiporter (xCT), sodium-coupled neutral amino acid transporter 1 (SNAT1), and sodium-coupled neutral amino acid transporter 2 (SNAT2). Gln enters cells through the coordinated action of ASCT2, ATB^0,+^, SNAT1, and SNAT2. Within the mitochondria, Gln is converted to Glu through GLS, and Glu is exported via the reverse transporter xCT in exchange for Cys. Both Cys and Glu serve as substrates for GSH synthesis, which is a crucial antioxidant molecule [12]. Notably, LAT1 plays a unique role in transporting Gln out of the cell and facilitates the entry of essential amino acids, such as Leu. This dual function is important because Leu activates mTOR, a central regulator of cellular metabolism and growth. The uptake and metabolism of Gln within tumor cells are illustrated in Fig. 1. In the tumor environment, the increased cellular demand for Gln is met by the upregulation of Gln transporter expression. Numerous studies have shown increased expression of Gln-related transporters in tumors [13]. Consequently, novel anticancer drugs may target these transporters.

## Expression and Role of Gln Transporter in DSMT

DSMTs pose a substantial global threat to human health, including colorectal cancer (CRC), gastric cancer (GC), esophageal cancer (EC), pancreatic cancer (PC), and liver cancer (LC). According to the Global Cancer Statistics 2020, DSMTs rank among the top 15 in both incidence and mortality rates [1]. These tumors are characterized by a propensity for metastasis and frequently diagnosed at advanced stages, contributing to elevated mortality rates. The traditional treatment modalities for DSMTs include radiotherapy, chemotherapy, surgical resection, and molecular-target medications [4,14,15]. Multiple studies have established a considerable correlation between Gln transporters and growth and proliferation of DSMTs. The expression patterns of Gln transporters in DSMTs are shown in Table 1.

**Table 1:** Glutamine transporters in tumors of the digestive system

Glutamine transporter	Gene	Transport substrates	Tumors overexpressing Gln transporter
ASCT2	*slc1a5*	Ala, Ser, Cys, Thr, Gln	CRC, GC, EC, PC, HCC
ATB^0, +^	*slc6a14*	Neutral and cationic amino acids	HCC
LAT1	*slc7a5*	Neutral amino acids	CRC, PC
LAT2	*slc7a8*	Neutral amino acids	PC
SNAT1	*slc38a1*	Neutral amino acids	HCC
SNAT2	*slc38a2*	Neutral amino acids	GC, PC
SNAT7	*slc38a7*	Gln, Ala, Ser, Asn	PC

Note: ASCT2: Alanine-serine-cysteine transporter 2; ATB^0,+^: Amino acid transporter B^0,+^ ; LAT1: L-type amino acid transporter 1; LAT2: L-type amino acid transporter 2; SNAT1: Sodium-coupled neutral amino acid transporter 1; SNAT2: Sodium-coupled neutral amino acid transporter 2; SNAT7: Sodium-coupled neutral amino acid transporter 7; Ala: Alanine; Ser: Serine; Cys: Cysteine; Thr: Threonine; Gln: Glutamine; Glu: Glutamate; Asn: Asparagine; CRC: Colorectal cancer; GC: Gastric cancer; EC: Esophagus cancer; PC: Pancreatic cancer; HCC: Hepatocellular carcinoma.

### ASCT2

ASCT2, alanine serine cysteine transporter 2, is encoded by *slc1a5*. It is a sodium-coupled transporter of neutral amino acids, such as alanine, serine, cysteine, and Gln, and when ASCT2 transports Gln into the cell, sodium ions enter synergistically [16]. ASCT2 plays a crucial role in determining the direction of Gln transport, based on the concentration gradient of these amino acids within the cell. In addition, it can transport certain neutral amino acids and Gln with low affinity. The transport activity of ASCT2 towards Gln is further promoted at low pH. ASCT2 exhibits a notably higher affinity for Gln compared with that of other neutral amino acids, underscoring its importance as one of the most critical transporters for Gln [17]. ASCT2 is primarily localized in various tissues including the lungs, skeletal muscle, colon, kidney, testis, brain, T cells, and adipose tissue [18].

As a key Gln transporter, ASCT2 plays a substantial role in enhancing intracellular Gln uptake [19]. Notably, ASCT2 is overexpressed in highly proliferative cancer cells to meet the increasing demand for Gln, making it a promising cancer target. Elevated ASCT2 expression was also observed in DSMTs. In a comprehensive study involving 90 patients with CRC, immunohistochemical analysis revealed a substantially higher expression of ASCT2 in CRC tissues than that in normal mucosal tissues. Silencing ASCT2 expression in the HT29 and HCT116 CRC cell lines through siRNA inhibited tumor cell proliferation and induced apoptosis [20]. Moreover, ASCT2 knockout in CRC cell lines leads to a substantial reduction in Gln uptake by over 60%, subsequently decreasing tumor growth [21]. CRC cells are sensitive to Gln deprivation, resulting in notable antiproliferative effects attributed to a decrease in intermediate metabolites in the TCA cycle and a substantial reduction in antioxidant levels [22]. High ASCT2 expression is considerably associated with *KRAS* mutations in CRC, and ASCT2 knockdown inhibits CRC cell growth [23]. These findings suggest that ASCT2 is a novel therapeutic target for CRC. Ye et al. [24] demonstrated high ASCT2 expression in the gastric tissues of patients with GC. ASCT2 knockdown in GC cells not only inhibited cell proliferation, but also induced G0/G1 cell cycle arrest and suppressed cell invasion. *In vivo* experiments further supported these findings, demonstrating a decrease in the relative tumor volume of xenograft tumors upon ASCT2 inhibition [25]. ASCT2 overexpression was also observed in EC tissues. Downregulation of ASCT2 in EC cells induces cell cycle arrest and apoptosis, and significantly reduces cell growth by blocking Gln transport [26]. Patients with high ASCT2 expression in esophageal squamous cell carcinoma were more prone to tumor invasion and metastases following surgical resection [27]. The overexpression of ASCT2 contributed to EC proliferation, migration, and Gln metabolism, thereby promoting the malignant phenotype of EC [28]. In patients with PC, ASCT2 expression is substantially higher than that in healthy pancreatic ductal cells [29]. ASCT2 is substantially upregulated in PC tissues, mediates Gln uptake into PC cells [30] and promotes cell proliferation, migration, and invasion [31]. ASCT2 silencing substantially inhibits PC growth, resulting in decreased Gln consumption, α-KG production, and ATP generation. ASCT2 knockdown induces apoptosis through the Akt/mTOR signaling pathway and attenuates tumor growth in xenograft mice bearing BxPC-3 tumors [32]. Early studies on LC using a human hepatocellular carcinoma cell line (Hep G2 cells) revealed that both *slc1a5* promoter activity and the protein expression of ASCT2 were dependent on the presence of Gln. The ASCT2promoter was activated when Gln was sufficient to upregulate ASCT2 expression, and deprivation of Gln decreased the cell growth rate and ASCT2 expression [33]. ASCT2 expression was increased in the liver tissues of patients with hepatocellular carcinoma (HCC) compared to that in healthy individuals, and high ASCT2 expression correlated with larger tumor sizes in patients with HCC. The degree of Gln dependency can vary among different cancer subtypes and even among individual cancer cell lines. For instance, in the liver tissues of patients with HCC, ASCT2 expression was found to be higher than that in healthy individuals. Moreover, high ASCT2 expression correlated with larger tumor sizes in patients with HCC. Notably, poorly differentiated hepatomas such as, SK-Hep, FOCUS, and PLC/PRF/5 are more glutamine-dependent than HepG2, Hep3B, and Huh-7 cells [34,35]. These findings highlighted ASCT2 as a promising therapeutic target for DSMTs.

*SLC1A5_var*, a short variant of the *slc1a5* gene lacking exon 1, functions as a mitochondrial Gln transporter. Overexpression of *SLC1A5_var* facilitated Gln-induced ATP production and GSH synthesis, and increased ROS levels, thereby playing a pivotal role in metabolic reprogramming in cancer. PC cells exhibited higher endogenous expression of the *SLC1A5_var* gene compared with that in healthy pancreatic ductal epithelial cells. Knockdown of *SLC1A5_var* resulted in reduced GSH and increased ROS levels, while overexpression of *SLC1A5_var* promoted PC cell and tumor growth [36]. Targeted modulation of mitochondrial *SLC1A5_var* is a crucial strategy for PC treatment.

### LAT1

LAT1, which is encoded by *slc7a5*, serves as a sodium-independent amino acid exchanger that facilitates the transport of large neutral amino acids. LAT1 is highly expressed in the brain, testes, ovaries, pancreas, and placenta, whereas it is downregulated in the lungs, heart, and liver [37].

Through transcriptional and metabolic reprogramming, LAT1 plays a crucial role in maintaining intracellular amino acid levels in response to *KRAS* activation. This protein operates by exporting Gln in exchange for other amino acids, meeting the heightened demand for protein synthesis in cells and forming the basis for the proliferation of *KRAS*-mutant cells. *KRAS*-mutant CRC cells specifically upregulate LAT1, and deletion of LAT1 impedes the growth and metastasis of CRC tumors [38]. LAT1 has been identified as a target gene of *miR-497-5p* and the overexpression of *miR-497-5p* inhibits CRC cell growth, invasion, and Gln metabolism. Conversely, LAT1 overexpression promotes CRC cell growth, invasion, and Gln metabolism [39]. In a study involving 97 patients with PC, 51 exhibited high LAT1 expression. The substantial correlation between LAT1 expression and tumor size suggests that LAT1 could serve as an important marker for PC treatment in clinical settings [40].

### ATB^0,+^

ATB^0,+^, encoded by *slc6a14*, belongs to the sodium-dependent neutral and cationic amino acid transport system and facilitates the transport of neutral and cationic amino acids. The superscripts 0 and + in ATB^0,+^ denote their ability to transport uncharged neutral amino acids (0) and positively charged cationic amino acids (+). Its substrates include all essential amino acids, including Gln. In contrast to ASCT2, ATB^0,+^ catalyzes unidirectional influx of substrates into cells [41]. Although it has a relatively weak affinity for Gln, ATB^0,+^ plays an auxiliary role in Gln transport [42]. ATB^0,+^ is expressed in various tissues, such as the lungs, trachea, salivary glands, stomach, colon, mammary glands, and hippocampus [43].

The rate of Gln uptake by human LC cells is several times higher than that of healthy liver cells. Amino acid inhibition, kinetics, northern blotting, RT-PCR, and restriction enzyme analysis identified ATB^0,+^ as the transporter responsible for Gln uptake in six human liver cancer cell lines. ATB^0,+^ mRNA is highly expressed in LC biopsies, but not in isolated normal liver cells [35]. In HCC cells, ATB^0,+^ and ASCT2 collectively account for the majority of Gln uptake. Stable transfection of SK-Hep LC cells with ATB^0,+^/ASCT2 antisense RNA-expressing plasmids resulted in a 73% reduction in ATB^0,+^/ASCT2 mRNA levels and a 65% reduction in the Gln transport rate, leading to a 98% reduction in cell number after 48 h. The expression of ATB^0,+^/ASCT2 is necessary for the growth and viability of SK-Hep cells [44]. Further exploration of the role of ATB^0,+^/ASCT2 as a target of other DSMTs could potentially provide selective targets for various DSMTs.

### SNAT1, SNAT2, and SNAT7

SNAT1, SNAT2, and SNAT7 are sodium-coupled neutral amino acid transporters encoded by *slc38a1*, *slc38a2* and *slc38a7*, respectively, and are predominantly expressed in the liver, skeletal muscle, brain, placenta and intestine [45].

Immunohistochemical studies on SNAT1 expression in GC tissues and adjacent healthy gastric tissues from patients with GC have revealed low or undetectable levels in healthy gastric tissues. In contrast, high SNAT1 expression was detected in 495 out of 896 GC samples. Inhibition of SNAT1 expression by siRNA resulted in reduced growth of the cultured GC cell line SH-10-TC [46], indicating that SNAT1 overexpression contributes to GC tumorigenesis by promoting cell proliferation. Inhibition of SNAT1 expression reduced cell migration. Xie et al. [47] demonstrated that the inhibition of SNAT1 using shRNA technology-reduced PC cell growth and migration. SNAT2 was more highly expressed in GC cells than in parental GC cells. Overexpression of SNAT2 substantially increased Gln depletion in GC cells, promoting GC cell stemness, which is dependent on Gln depletion [48]. SNAT2 is crucial for amino acid accumulation, maintenance of cellular osmotic pressure, and activation of mTORC1. It also provides Gln for catabolism, making it a potential therapeutic target for cancer treatment. Gauthier-Coles et al. [49] used a SNAT2 inhibitor to suppress the proliferation and growth of a PC cell line. SNAT7, a local lysosomal transporter, exports Gln from the lysosomes into the cytoplasm. Meng et al. demonstrated that Gln induces cell growth and proliferation in PC cells, and deletion of SNAT7 substantially inhibits cellular mTORC1 activation, cell growth, and proliferation [50].

## Drugs Targeting Gln Transporters in the Treatment of the Digestive System Tumors

The concept of restricting Gln to treat tumors has been long standing [51,52]. Proliferation and growth of DSMTs are closely linked to various Gln transporters. Inhibitors targeting these transporters can hinder Gln transport, thereby offering a potential avenue for tumor therapy. The relevant therapeutic agents are summarized in Table A1.

### Gln analogues

L-γ-Glutamyl-p-nitroanilide (GPNA) serves as a structural analog of Gln, competitively inhibiting ASCT2 [53]. GPNA hinders the growth of PC and HCC cells by suppressing the uptake of Gln via ASCT2 [32,54]. Furthermore, GPNA inhibits the transport activity of *SLC1A5_var* [36]. GPNA (rASCT2 IC_50_ = 70.2 μM; hASCT2 IC_50_ = 1.2 mM) is recognized as a commercially available competitive inhibitor of rat ASCT2 transporter. However, GPNA inhibits LAT1- and LAT2-mediated Gln uptake in a various human-derived cancer cells, and GPNA hydrolysis by the γ-glutamyltransferase enzyme generates the cytotoxic metabolite para-nitroaniline [55]. Currently, the lack of specificity and toxicity of GPNA toward ASCT2 limits its use in basic research. Researchers have performed structural modifications of Gln analogs to analyze the structure-activity relationship of different Gln modifiers as ASCT2 inhibitors. They found that Gln 2-substitution was the determinant of ASCT2 activity across all positional substitutions. In 2018, Schulte et al. synthesized V-9302 (rASCT2 IC_50_ = 9.0 μM; hASCT2 IC_50_ = 9.6 μM), a small molecule competitive antagonist of Gln that exhibits highly selective inhibition of ASCT2. Compared with GPNA, V-9302 is 100 times more potent in inhibiting ASCT2 and is currently in preclinical studies. V-9302 inhibited Gln uptake by ASCT2 cells, leading to attenuated growth and proliferation, increased apoptosis, and increased oxidative stress in CRC cells [56]. Jin et al. [57] discovered that the combined use of V-9302 and the GLS inhibitor, CB-839, induced apoptosis in HCC and enhanced tumor suppression in an HCC xenograft mouse model. The dual inhibition of Gln metabolism by targeting the Gln transporters ASCT2 and GLS may represent a novel potential therapeutic strategy for HCC. However, Bröer et al. [58] reported that V-9302 inhibited LAT1- and SNAT2-mediated Gln uptake, but not ASCT2-mediated Gln uptake. Therefore, further investigation is required to elucidate the mechanism by which V-9302 inhibits Gln uptake.

### ASCT2 inhibitors

Lobetyolin, a polyacetylene glucoside derived primarily from the roots of *Codonopsis pilosula*, induces apoptosis by inhibiting ASCT2-mediated Gln uptake, thereby suppressing GC [59]. Berberine, a major component of various traditional Chinese herbs, exhibits antioxidant, anti-inflammatory, and antimicrobial properties. Berberine inhibits cancer cell proliferation and induces apoptosis. Zhang et al. [60] found that berberine reduced the expression of ASCT2 in LC cells by regulating c-Myc, leading to the suppression of Gln uptake and the subsequent inhibition of LC cell proliferation. Resveratrol reduced Gln and GSH uptake by downregulating ASCT2 expression in LC. Combined treatment with cisplatin substantially increased ROS production, resulting in stronger antitumor effects [61]. Topotecan, a camptothecin derivative, exerts substantial anticancer effects on GC by reducing Gln uptake through downregulation of ASCT2 expression. This induces oxidative stress and activates mitochondrial apoptosis [62]. In CRC cells, the mutant isocitrate dehydrogenase 1 inhibitor Ag120 inhibited ASCT2-mediated Gln uptake, leading to decreased cell proliferation and increased oxidative stress both *in vitro* and *in vivo* [63]. Galectin-12, a β-galactoside-binding protein with multiple functions, including the regulation of cell apoptosis, growth, and differentiation, was reported by Katzenmaier et al. to substantially reduce Gln uptake in CRC cell lines by binding to ASCT2 [64]. The novel Na^+^/K^+^-ATPase inhibitor RX108 substantially downregulates ASCT2 protein expression and reduces Gln levels in HCC cells and tumors. RX108 also diminishes energy metabolism in HCC cells, including GSH, NADH, NADPH, and the mitochondrial respiratory oxygen consumption rate [65].

### ATB^0,+^ inhibitors

Alloferon is a peptide isolated from the immune system of insects and is extracted from the blood of periwinkle larvae after a bacterial attack. It possesses antitumor properties. Jo et al. demonstrated that alloferon substantially decreases the expression of ATB^0,+^ in PC cells and reduces ATB^0,+^-mediated Gln uptake, which in turn inhibits PC cell growth and proliferation [66].

### LAT1 inhibitors

The inhibition of LAT1 can suppress the growth of tumor cells, therefore, LAT1 inhibitors have garnered considerable attention as potential anticancer drugs. 2-Amino-2-norbornanecarboxylic acid (BCH), a non-metabolizable leucine analog, inhibits the activity of LAT1, leading to the depletion of neutral amino acids essential for tumor cell growth, consequently inducing tumor cell apoptosis [67]. BCH is an inhibitor of the amino acid transport system *L*, affecting LAT1, LAT2, LAT3, and LAT4. The drawbacks of using BCH as an LAT1 inhibitor are its low selectivity and low affinity, which require high concentrations to exert inhibitory effects [68]. JPH203, a selective LAT1 inhibitor and tyrosine structural analogue [69], has shown promising results. Treatment with JPH203 substantially inhibited the growth of GC and CRC cells, effectively suppressing CRC growth and metastasis in mouse models, while sparing EC cells [70,71]. Importantly, JPH203 did not harm the healthy cells [72]. The first-in-human Phase I clinical trial of JPH203 in patients with advanced solid tumors demonstrated improved therapeutic effects, particularly in two patients with CRC treated daily with intravenous JPH203 [73]. JPH203 is anticipated to become a new antitumor drug in clinical practice. SKN103, another LAT1 inhibitor, suppresses the growth of PANC-1 cells, and its combination with cisplatin enhances growth inhibition of cancer cells [74]. To develop effective antitumor drugs targeting LAT1, it is crucial to generate compounds with high affinity and selectivity for LAT1. These compounds specifically inhibit LAT1 without affecting other transporters expressed in noncancerous cells, thus minimizing unintended side effects.

### SNAT2 inhibitors

3-(N-Methyl(4-methylphenyl)sulfonamido)-N-(2-trifluoromethylbenzyl)thiophene-2-carboxamide (MMTC) is a novel SNAT2 inhibitor that effectively inhibits the growth and proliferation of PC cells and mTORC1. The combination of MMTC and the Bay-876 glucose transporter inhibitor successfully inhibited the proliferation of PC cell lines. Furthermore, combining different transporter inhibitors enhances therapeutic efficacy, showing the potential for synergistic effects in the treatment of pancreatic cancer [49].

### ASCT2 monoclonal antibodies

Antibody therapy is developing rapidly, with monoclonal antibodies proving to be more specific and stable than traditional small-molecule inhibitors. In a study by Suzuki et al., monoclonal antibodies KM4008, KM4012 and KM4018 were generated to recognize structural domains on the surface of ASCT2 cells. These antibodies were developed using *slc1a5*-expressing Chinese hamster ovarian cells as immunogens. All three antibodies inhibited the Gln-dependent WiDr colon cancer cell proliferation *in vitro* [75]. Another novel humanized anti-ASCT2 monoclonal antibody, KM8094, exhibited anti-tumor activity in GC patient-derived xenografts. KM8094 effectively inhibits Gln uptake, induces antibody-dependent cytotoxicity in GC cells *in vitro*, and inhibits the growth of various GC cell-transplanted tumors *in vivo* [76]. These findings highlight the potential of using monoclonal antibodies to target Gln transporters for the treatment of DSMTs.

### Nanoparticle approach for drug delivery

Utilizing nanoparticles as carriers for covalently bonded pharmaceuticals is an increasingly popular strategy for enhancing drug delivery. Various methods have been explored, including transporters. For instance, tyrosine-modified irinotecan-loaded liposomes have been developed to simultaneously target LAT1 and ATB^0,+^ for potent therapy of BxPC-3 cells. These liposomes demonstrate superior cytotoxicity, apoptotic properties, and enhanced antitumor activity [77]. Another approach involves the development of actively targeting liposomes functionalized with lysine and a polyoxyethylene stearate conjugate loaded with docetaxel. This actively targeted nano-preparation efficiently delivered docetaxel to HepG2 cells, resulting in high antitumor efficacy and low systemic toxicity [78]. Active targeting is achieved by adding a ligand specific to overexpressed substances on the cell surface or intracellular surface of the targeted cell to the surface of the vector. In addition to active targeting, light-sensitive nanoparticles have gained attention because of their ability to provide remote control of light-triggered drug release. This feature is particularly important for improving tumor suppression while minimizing drug damage to healthy tissues. External light triggering allows the precise spatiotemporal control of drug release. Light-sensitive nanoparticles and ASCT2 inhibitors have been investigated for the treatment of breast cancer [79], and their applications in DSMTs should be explored in future studies.

## Conclusion and Discussion

Over the past decade, Gln has emerged as one of the most extensively studied substances involved in cancer cell metabolism. In DSMTs, Gln serves as a crucial substrate for tumor cell energy, acting as a precursor for nucleotide, lipid, protein, and GSH synthesis. These components are essential for meeting the energy and biosynthetic demands of tumor cells, thus facilitating their malignant progression. Several Gln transporters, including ASCT2, LAT1, LAT2, ATB^0,+^, SNAT1, SNAT2, and SNAT7 play pivotal roles in Gln uptake and utilization. LAT1, LAT2, ATB^0,+^, SNAT1, SNAT2, and SNAT7, with particular emphasis on ASCT2, are potential target for therapeutic intervention in DSMTs by modulating Gln transport.

However, current studies targeting Gln transporters for the treatment of DSMTs remain limited, especially clinical data. Many studies have been limited to patient immunohistochemistry experiments, lacking more in-depth clinical investigations. Among the drugs targeting Gln transporters for DSMTs treatment, only the LAT1 inhibitor JPH203 has entered clinical trials. Other inhibitors have faced challenges, such as poor specificity or high toxicity, which prevent their progression to clinical trials. The importance of the various Gln transporters in DSMTs varies. For example, ASCT2 facilitates the uptake of Gln into the cells, whereas LAT1 is responsible for transporting Gln out of the cell in exchange for leucine, thereby activating mTOR. In addition, the specific roles of Gln transporters may vary among different cancer types, although they collectively contribute to the heightened demand for Gln. ASCT2 plays a crucial role in mediating Gln metabolism and maintaining redox homeostasis in PC cells. In contrast, in CRC, elevated ASCT2 expression is substantially linked to *KRAS* gene mutations. ASCT2 is the most important Gln transporter, and frequently overexpressed in various cancer types, including CRC and PC. This overexpression is associated with an increased demand for Gln to support rapid cell division and energy production. There is a correlation between elevated ASCT2 expression and aggressive tumor phenotypes in CRC and PC. These findings suggest that ASCT2 contributes to the aggressiveness and progression of these cancers. ASCT2 has emerged as a promising therapeutic target and ongoing research is focused on developing inhibitors that can selectively target this transporter. Inhibition of ASCT2 holds the promise of disrupting the Gln supply to cancer cells, thereby impeding their growth and survival. Among the ASCT2 inhibitors, GPNA, berberine, resveratrol, topotecan, and the galactoglucan lectin-12 are in the *in vitro* experimental stage, whereas V-9302, Lobetyolin, Ag120, and RX-108 are in the *in vivo* animal experimental stage. GPNA and V-9302, which are structural analogs of Gln, were effectively inhibited. However, GPNA’s high toxicity of GPNA limits its use in cellular experiments, and the mechanism of V-9302 inhibition is controversial and requires further investigation [80]. Natural drugs, such as lobetyolin, berberine, resveratrol, and topotecan effectively inhibit the growth and proliferation of DSMTs. The conformational relationship between their structure and ASCT2 inhibition needs to be further investigated for the potential discovery of specific ASCT2 inhibitors from traditional Chinese medicine and natural drugs. When treating DSMTs with ASCT2 inhibitors, it is crucial to recognize that although ASCT2-mediated Gln uptake is inhibited, other transporters, such as SNAT1 and SNAT2, may still facilitate Gln transport. Therefore, employing a combination of Gln transporter inhibitors may be a more effective approach for tumor suppression. Gln transporters have been identified as prognostic biomarkers and/or predictors of cancer treatment efficacy in certain tumors, suggesting that future studies should focus on the role of transporters in tumors to aid in the clinical identification of tumor stage and prognosis. The discovery of more targeted inhibitors that can spare healthy cells could substantially improve the clinical treatment of DSMTs.

Substantial effort has been devoted to developing new inhibitors of Gln transporters; however, to date, only JPH203 has progressed to clinical trials. This suggests that there is still a considerable distance to cover in this field. Drug specificity is a critical consideration in targeted therapies and the combination of structural biology and medicinal chemistry plays a pivotal role in creating highly specific drugs. One obstacle hindering the development of new inhibitors may be the unclear molecular and structural basis of human Gln transporters. The 3D structures of some transporters, such as ATB^0,+^, SNAT1, SNAT2, and SNAT7, have not yet been suitably analyzed. The transporter structure is vital for understanding its interaction with drugs, making the study of its 3D structure as important as its clinical relevance at a functional level. Natural products are integral to tumor therapy, and the exploration of functional basic structural units from natural products, along with their structural modifications, can be an effective approach for developing antitumor drugs. Some inhibitors face challenges in *in vivo* testing because of their poor specificity. One solution for the poor specificity is to employ nanoparticle-loaded drugs for delivery to enhance bioavailability, achieve drug accumulation in specific organs or tissues, and reduce toxic side effects. Moreover, combination therapies targeting multiple tumor therapeutic targets may be more effective than monotherapies. Ongoing research focuses on the tissue- and cell-specific expression, regulatory mechanisms, and structure-activity relationships of Gln transporters. The aim is to discover more effective and targeted inhibitors for the treatment of digestive tumors.

## Data Availability

No data were used for the research described in this study.

## Appendix A

**Table A1:** Drugs that target glutamine transporters in the treatment of tumors of the digestive system

Drugs	Target	Combination	Cancer	Cell line /experimental model	Mechanism	References
MMTC	SNAT2	Bay-876	Pancreatic cancer	HPAFII	Inhibit the growth and proliferation of pancreatic cancer cells and inhibit mTORC1	[49]
GPNA	ASCT2		Pancreatic cancer	BxPC-3/BALB/C nude mice bearing BxPC-3 xenograft tumors	Structural analogue of glutamine; competitively inhibits ASCT2	[32,54]
			Hepatocellular carcinoma	HepG2		
V-9302	ASCT2		Colorectal cancer	HCT116, HT29, Colo205, RKO	Structural analogue of glutamine; competitively inhibits ASCT2	[56]
		CB-839	Liver cancer	Hep3B, Huh7, HepG2, SNU398, SNU449, Huh6, SK-Hep1, JHH1, SNU387, PLC/PRF/5, MHCC97H/BALB/C nude mice bearing SNU398 and MHCC97H xenograft tumors	Structural analogue of glutamine; competitively inhibits ASCT2	[57]
Lobetyolin	ASCT2		Colorectal cancer	HCT-116/nude mice bearing HCT116 xenograft tumors	Inhibits glutamine uptake via ASCT2	[59]
Berberine	ASCT2		Liver cancer	Hep3B, BEL-7404/ BALB/C nude mice bearing Hep3B xenograft tumors	Reduces the expression of ASCT2 and inhibits glutamine uptake	[60]
Resveratrol	ASCT2	Cisplatin	Hepatocellular carcinoma	C3A, SMCC7721	Reduces the expression of ASCT2 and inhibits glutamine uptake	[61]
Topotecan Hydrochloride	ASCT2		Gastric cancer	BGC-823, MGC-803	Reduces the expression of ASCT2 and inhibits glutamine uptake	[62]
Ag120	ASCT2		Colorectal cancer	HCT116, HT29/ BALB/C nude mice bearing HCT116 xenograft tumors	Inhibits glutamine uptake via ASCT2	[63]
Galectin-12	ASCT2		Colorectal cancer	HCT116-Tet-On, HCT116-Gal12	Competitively inhibits ASCT2	[64]
RX108	ASCT2		Hepatocellular carcinoma	Huh7, Hep3B/nude mice bearing Hep3B xenograft tumors	Reduces the expression of ASCT2 and inhibits glutamine uptake	[65]
Alloferon	ATB^0,+^		Pancreatic cancer	Panc-1, AsPC-1	Reduces the expression of ATB^0,+^ and inhibits glutamine uptake	[66]
JPH203	LAT1		Esophageal cancer, gastric cancer, colorectal cancer	TE5, TE6, TE14, MKN1, MKN45, Lovo, HT29/ BALB/C nude mice bearing CT26 xenograft tumors	Tyrosine structural analogue, inhibits LAT1 activity, effectively inhibits the growth and metastasis of tumor cells	[70–71]
SKN103	LAT1		Pancreas ductal adenocarcinoma	PANC-1 , MIAPaCa-2	Competitively inhibits LAT1	[74]
KM8094	ASCT2		Gastric cancer	SNU-16, AGS, NCI-N87/SCID mice bearing xenografts from the gastric cancer cell lines (HSC-39, SNU-16, HSC-40A, MKN28, 60As6, and HSC-60)	Inhibits glutamine uptake	[76]
